# New Marker of Brain–Heart Interaction: Tpeak–Tend Interval

**DOI:** 10.3390/medicina62040695

**Published:** 2026-04-04

**Authors:** Nazire Belgin Akilli, Huseyin Mutlu, Zerrin Defne Dundar, Omer Ozberk, Ramazan Koylu, Yahya Kemal Gunaydın, Basar Cander

**Affiliations:** 1 Emergency Department, Konya Training and Research Hospital, Konya 42090, Turkey; hmutlu70@hotmail.com (H.M.); zerdef@hotmail.com (Z.D.D.); drkoylu@yahoo.com (R.K.); gsykg@yahoo.com (Y.K.G.); basarcander@yahoo.com (B.C.); 2 Radiology Department, Konya Training and Research Hospital, Konya 42090, Turkey; omerozberk@gmail.com

**Keywords:** brain–heart interaction, stroke, Tpeak–Tend, ECG, QT

## Abstract

*Background and Objectives*: The interaction between the brain and heart has become more interesting in the last 20 years. The most common cardiac complications after stroke are myocardial infarction, heart failure, arrhythmias, electrocardiographic disturbances, repolarization disorders, and sudden cardiac death. The prolonged Tpeak–Tend interval is an indicator of the electrical heterogeneity of the myocardium (abnormal repolarization) that causes malignant arrhythmias. We aimed to investigate whether the Tpeak–Tend interval, which reflects the heterogeneity of repolarization, is prolonged in stroke and its relationship with short-term mortality. *Materials and Methods*: Individuals over the age of 18 who presented with hemorrhagic or ischemic stroke were included in the study. Demographic characteristics, laboratory and imaging findings of the patients were recorded. ECGs were obtained at the time of admission to the hospital and 24 h later. Patients were followed for in-hospital mortality. *Results*: 89 (82.4%) of the patients had ischemic stroke, 19 (17.6%) had hemorrhagic stroke. It was determined that Tp-eV2 and Tp-eV5 at hospital admission were significantly longer than the 24th hour values. A total of 92.01 (16.3) ms at Tp-eV2 admission, 84.1 (16.3) ms after 24 h (*p* = 0.003), 91.9 (7.3) msTp-eV5 at admission, and 81.6 (17.8) ms (*p* = 0.000) after 24 h. In multivariate logistic regression analysis of in-hospital mortality, Tp-eV2 (HR: 0.96 (95% CI 0.93–0.99) *p* = 0.008) was determined as an independent predictor among cardiovascular parameters. *Conclusions*: Tp-e intervals were prolonged in both leads V2 and V5 in patients with stroke. Prolongation of lead V2 in the Tp-e interval is an independent indicator of short-term mortality among cardiovascular parameters.

## 1. Introduction

Since the early 2000s, the interaction between the brain and heart has attracted more attention from scientists. When the studies are reviewed, it is seen that this interaction is bidirectional with different pathways. The risk caused by cardiac pathologies in ischemic stroke etiology is known to all physicians. However, the interaction between these two organs is not limited to that risk. For example, Kim et al. have reported that low cardiac output impairs cerebral autoregulation and cerebral hypoperfusion worsens cognitive functions in patients with heart failure [1]. By a reverse pathway, it has been reported that the brain affects the regulation of sympathetic activation and fluid hemostasis in patients with heart failure, resulting in worsening of symptoms in heart failure and left ventricular remodeling [1].

Most of the studies investigating the brain–heart axis have focused on heart diseases developing after ischemic or hemorrhagic stroke. It is known that the contribution of cardiac complications developing after stroke to mortality varies between 12.5% and 22.7% [2]. The most serious cardiac complications occur within the first 14 days after stroke [3]. These complications are myocardial infarction, congestive heart failure, hemodynamic instability, left ventricular systolic dysfunction, diastolic dysfunction, arrhythmias, electrocardiographic disorders, repolarization disorders, and sudden cardiac death [2,4].

T waves represent ventricular repolarization in the electrocardiogram (ECG). The prolonged Tpeak–Tend interval is an indicator of the electrical heterogeneity of the myocardium (abnormal repolarization) that causes malignant arrhythmias and is associated with a 1.14-fold increased risk of ventricular tachycardia/ventricular fibrillation, sudden cardiac death, cardiovascular system, and all-cause cardiac death [5,6]. In our study, we aimed to investigate whether repolarization disorder occurring in stroke causes the prolongation of the Tp-e interval and its relationship with short-term mortality in stroke patients.

## 2. Materials and Methods

### 2.1. Study Population

This study was conducted in the emergency department (ED) of an 800-bed academic regional hospital in Konya that provides third-level health services. This ED serves approximately 220,000 patients per year. Patients over the age of 18 who presented with ischemic and hemorrhagic stroke over a one-year period were included in the study. Informed consent was obtained from the patients themselves and, in cases where it was not possible, from their legal guardian. Patients under 18 years of age, patients whose ECG cannot be taken after 24 h, pregnant patients, patients with electrolyte abnormalities and using drugs that prolong QT, and patients who did not give their consent were excluded from the study. Ethics committee approval was obtained from the local ethics committee prior to the study, and the study was in accordance with the Helsinki declaration.

### 2.2. Study Protocol

The demographic characteristics, vital signs, and medical history of patients were recorded. Glasgow Coma Score was calculated. ECGs were performed at hospital admission and 24th hour of admission. The ordered complete blood count, troponin, and biochemical parameters during admission to the ED were recorded.

Brain computed tomography and diffusion magnetic resonance images taken at the ED admission were evaluated by an experienced radiologist, unaware of the patient’s clinical information. Stroke type (hemorrhagic, ischemic), lesion side, lesion location (tentorial, supratentorial), insular cortex, and thalamus involvement were examined. The Cavalieri method was used in the calculation of lesion volume [7]. The sum of the surface areas of the obtained sections was multiplied by the distance between the sections, that is, the section thickness. This process is expressed by the formula below:

Volume (cm^3^) = t × (a_1_ + a_2_ + … + a_n_)

where t is the thickness, and a is the area.

Echocardiography was performed using the routine echocardiography protocol within the first 24 h. Ejection fractions were recorded. The patients were followed up by the hospital information system in terms of in-hospital mortality.

### 2.3. ECG Measurements

ECGs were performed in all patients at ED admission and at the 24th hour with Nihon Kohden ECG 1250 Cardiofax S (2009 Tokyo, Japan) device with 25 mm/s velocity and 10 mm/1 mV amplitude. The ECGs were scanned at a 600-dpi resolution, and measurements were made by electronic cursor from the screen by two experts who were blinded to the clinical status of the study population. ST elevation, ST depression, and T negativity seen in the ECGs were recorded, and QT intervals and Tp-e intervals were measured. Ischemic ST-T wave changes were defined as new ST-segment elevation (1 mm), ST-segment depression (0.5 mm), and T wave inversion (2 mm) observed in two consecutive leads [8].

**Figure 1:**
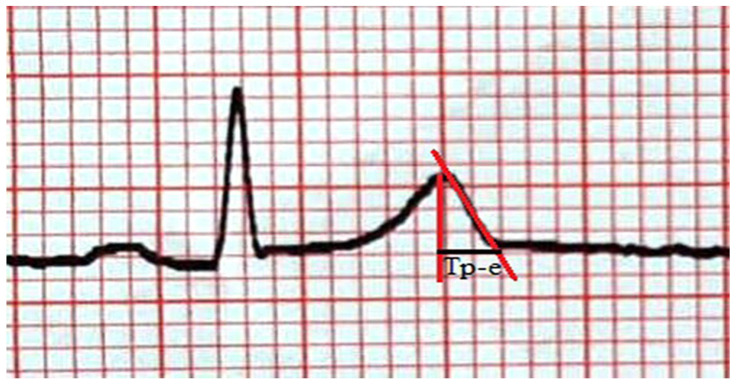
Measurement of the Tp-e interval by the tangent method.

The QT interval was measured as the distance between the beginning of the QRS and the end point of the T wave in derivations V2 and V5. The QTc was calculated using the Bazett formula. The Tp-e interval was measured in V2 and V5 derivations by the Tangent method [9]. The point where the line passing tangent from the peak of the T wave to the downward slope of the T wave intersects the isoelectric line was marked. If the T wave is negative or biphasic, this line was drawn by marking the lowest point of the T wave. If the T wave is followed by the U wave, the lowest point between the T and U waves was taken as the end of the T wave. By measuring the distance between these two points on the isoelectric line, the Tp-e interval was calculated (Figure 1). The intra- and inter-observer variability of the Tp-e interval were 4.6% and 7.3%, respectively.

### 2.4. Statistical Analysis

Statistical analyses were performed using SPSS version 15.0 software. The conformity of the variables to normal distribution was examined by visual and analytical methods. Descriptive analyses were expressed as mean (standard deviation) for normally distributed variables, median (interquartile range) for non-normally distributed variables, and *n* (%) for categorical variables. In comparisons of two independent groups, Student’s t test was used for normally distributed variables, Mann–Whitney U test for non-normally distributed variables, and chi-square and Fisher test for categorical variables. In the comparison of two dependent groups, the paired Student’s test was used for normally distributed data, and the Wilcoxon test was used for non-normally distributed data. Univariate and multivariate logistic regression analyses were performed to examine the independent relationships of cardiovascular parameters affecting short-term mortality. A *p*-value of <0.05 was considered statistically significant.

## 3. Results

A total of 108 patients were included in the study. Forty-nine (45.4%) patients were male, and the median age of patients was 72.3 (10.5) years. The demographic characteristics of patients are shown in Table 1. Eighty-nine (82.4%) of the patients had ischemic stroke, and 19 (17.6%) of them had hemorrhagic stroke.

There was no significant difference between the calculated QTV2, QTV5, and pulse rate in the ECGs performed at admission and at 24th hour (at admission 342.0 (40.8) ms, 353.7 (45.4) ms, 81.0 (22.0)/min; at 24th hour 345.4 (44.4) ms, 349.0 (46.4) ms, 84.2 (21.0)/min; *p* = 0.61, *p* = 0.39, *p* = 0.25; respectively). It was determined that Tp-eV2 and Tp-eV5 at hospital admission were significantly longer than the 24th hour values (for Tp-eV2 92.01 (16.3) ms vs. 84.1 (16.3) ms, *p* = 0.003; for Tp-eV5 91.9 (17.3) ms vs. 81.6 (17.8) ms, *p* < 0.001).

In comparison, according to the stroke type, Tp-eV2 median (IQR) was 91 (29) ms and Tp-eV5 was 95 (24) ms in patients with ischemic stroke, while Tp-eV2 was 87 (15.5) ms and Tp-eV5was 93 (20) ms in patients with hemorrhagic stroke (*p* = 0.93 and *p* = 0.96, respectively). Tp-eV2 was 94.3 (16.9)ms, and Tp-eV5 was 93.2 (19.8) ms in 50 patients with insular cortex involvement. Tp-eV2 was 92 (16.9) ms and Tp-eV5 was 93.5 (22.2) ms in the group without insular cortex involvement (*p* = 0.5 and *p* = 0.93, respectively). However, Tp-eV2 was 95.6 (17) ms, and Tp-eV5 was 85.0 (15.7) ms in patients with right insular lesions. Tp-eV2 was 92.5 (17.1) ms and Tp-eV5 was 105.0 (19.4) ms in patients with left insular lesions (*p* = 0.55 and *p* = 0.001, respectively). Tp-eV2 was 84 (16.5) ms and Tp-eV5 was 91 (30.5) ms in 19 patients with thalamus involvement, while Tp-eV2 was 94 (21) ms and Tp-eV5 was 95 (29) ms in patients without thalamus involvement (*p* = 0.14 and *p* = 0.63, respectively). Tp-eV2 was 89 (22) ms and Tp-eV5 was 95 (26) ms in patients with supratentorial lesions, whereas Tp-eV2was 95 (17) ms and Tp-eV5 was 91 (20) ms in patients with infratentorial lesions (*p* = 0.79 and *p* = 0.78, respectively). Tp-eV2 was 91.4 (17.1) ms and Tp-eV5 was 86.3 (18) ms in right-sided lesions, Tp-eV2 was 94.8 (16.7) ms and Tp-eV5 was 101 (19.1) ms in left-sided lesions (*p* = 0.31 and *p* = 0.04, respectively). No correlation was found between troponin levels and TpeV2, TpeV5, TpeV2-24, and TpeV5-24. (*p* > 0.05).

Tp-eV2 was 100 (17.2) ms and Tp-eV5 was 98.3 (22.6) ms in patients who died in hospital, whereas Tp-eV2 was 89.9 (15.9) ms and Tp-eV5 was 91.6 (17.6) ms in survivors (*p* = 0.005 and *p* = 0.12, respectively).

Parameters with *p* < 0.20 in univariate analysis were included in the logistic regression model, which was performed to evaluate the independent relationships of cardiovascular parameters affecting short-term mortality. In multivariate logistic regression analysis, Tp-eV2 alone (OR: 0.96 (95% CI 0.93–0.99), *p* = 0.008) was found as an independent predictor for in-hospital mortality among cardiovascular parameters (Table 2).

## 4. Discussion

This study is a prospective cohort study investigating whether the Tp-e interval measured in leads V2 and V5 is prolonged with stroke and its relationship with short-term mortality in stroke patients. According to our study results, Tp-e intervals measured at admission to the hospital in stroke patients were prolonged in both leads, V2 and V5. The prolongation of the Tp-e interval in lead V2 was an independent predictor of short-term mortality among cardiovascular parameters. The Tp-e interval did not differ with stroke type, lesion location, insular cortex involvement, or thalamus involvement. However, the Tp-e interval in lead V5 of stroke patients with the left insular lesion was 20 ms longer than that of stroke patients with the right insular lesions. Additionally, the Tp-e interval in lead V5 is 15 ms longer in left-sided brain injury than in right-sided brain injury.

Patients with stroke become susceptible to possible cardiac complications as brain damage can alter autonomic and neurohumoral pathways involved in maintaining heart functions [4]. The most serious complications after ischemic stroke have been described in the acute phase [10]. The more severe stroke has a greater risk of developing cardiac complications [4]. At least one cardiac adverse event was reported in 19% of patients within the first 3 months after acute ischemic stroke [11]. Most sudden deaths after stroke are sudden cardiac deaths caused by fatal cardiac arrhythmia [12]. Likewise, the development of cardiac complications is associated with worse neurological results and 90-day disability [4,13]. Even if there is no structural heart disease in patients with both ischemic and hemorrhagic stroke, electrocardiographic changes are observed in more than 90% of patients in the acute phase [14]. It is suggested that most of these abnormalities are caused by autonomic dysfunction rather than a coronary artery disease association [15]. A multivariate analysis with the outcome of neurological functional improvement showed that atrial fibrillation, atrioventricular block, ST depression, ST elevation, and T wave inversion were independent risk factors [16].

Brain–heart interaction is bidirectional and the pathophysiological mechanisms affecting this axis are sympathetic system activation, hypothalamic–pituitary–adrenal (HPA) axis, immune and inflammatory responses, and intestinal dysbiosis [4].

Catecholamine storm may occur due to the activation of the HPA axis [11]. The most likely cause of cardiac damage after stroke is increased catecholamine levels, which cause impaired systolic and diastolic dysfunction, abnormal repolarization, and myocardial damage [17,18]. Sudden calcium increase caused by catecholamines secreted from both circulatory and myocardial nerve endings causes myofibrillar degeneration, myocardial cell necrosis, apoptosis, and myocytolysis and leads to ECG changes and systolic dysfunction [11,19]. ECG changes are more prominent in the anterior and lateral wall leads in the presence of catecholamine release [11]. In our results, Tp-eV2 was 8 ms, and Tp-eV5 was 11 ms longer at hospital admission than at the 24th hour. The prolongation of the Tp-e interval may be related to abnormal repolarization caused by activation of the HPA.

Studies with imaging methods show that the autonomic system is controlled by the cortical and subcortical network, including the bilateral insular cortex, anterior cingulate gyrus, amygdala, and hypothalamus, in addition to the level of the spine and brain stem [12]. It is accepted that the right insula is responsible for the control of the sympathetic tone and the left insula is responsible for the control of the parasympathetic tone. Bradycardia and asystole have been reported in right insular lesions and support the idea that right insular lesions cause parasympathetic overactivity [12]. In contrast, left insular lesions have been shown to cause increased sympathetic activity, and they are related to tachycardia and hypertension [20]. In the absence of coronary artery disease, it has been shown that an increase in sympathetic system activity increases the repolarization time [21]. In our results, the Tp-e interval in lead V5 was 20 ms longer in the group with left insular lesion compared to those with right insular lesion. It has been stated that left insular lesions change the autonomic balance in favor of the sympathetic system, and this may create a proarrhythmic situation [20]. The prolongation of the Tp-e interval in lead V5 suggests that this ECG parameter may be related to the sympathetic system. Another interesting aspect of the relationship between the autonomic nervous system and the heart is the ‘lateralization theory’ [20]. The nerve conduction from the brain to the heart is mainly ipsilateral. Right-side nerves innervate the right ventricle and left-side nerves innervate the left ventricle [22]. In our results, the Tp-e interval was 15 ms longer in the lead V5, reflecting the left ventricle, in the left hemispheric lesions compared to the right side. Left-sided damage may have caused sympathetic activation and thus repolarization disorder, predominantly in the left ventricle. This can be manifested by the prolongation of the Tp-e interval in the left-sided leads of the ECG.

Both HPA axis activation and sympathetic system activation cause prolongation of repolarization. ST changes, QT, and QTc intervals are used in clinical practice to detect repolarization anomalies. Therefore, studies on patients with stroke have focused on QT and QTc intervals. Stead et al. detected prolonged QTc in the ECGs of 35.7% of patients with stroke. The QTc interval longer than 438 ms in men and 440 ms in women was associated with a low 3-month survival rate and poor neurological outcome [23]. We also performed univariate analysis to determine cardiac parameters that affect in-hospital mortality among the cardiovascular parameters used in daily clinical practice. We included the history of coronary artery disease, hospital admission systolic and diastolic blood pressure, pulse, troponin, ejection fraction, ST depression, ST elevation, T wave inversion, QT, QTc, and Tp-e intervals measured at leads V2 and V5 in this analysis. In our multivariate analysis, Tp-eV2for in-hospital mortality was determined as an independent predictor. Although both parameters are indicative of repolarization disorder, the mechanisms of short-term cardiovascular complications may differ. Among the cardiac parameters used in daily clinical practice, Tp-eV2 is more valuable for short-term mortality and is associated with a 96% risk increase in in-hospital mortality alone.

### Limitations

A small sample size is a fundamental limitation that affects both the methodological robustness of a study and the interpretability of its findings in multiple ways. First, small samples may not adequately reflect the heterogeneity of the target population in terms of clinical, demographic, and prognostic variables. This situation increases the risk of selection bias, potentially leading to the individuals included in the study being systematically different from the real-world population. Consequently, the external validity and generalizability of the findings are significantly limited. Second, the single-center design of the study may fail to adequately represent a broader population in terms of demographic characteristics, disease spectrum, socioeconomic status, and referral patterns. This situation limits the external validity of the findings and their generalizability to different geographic regions and healthcare systems. Furthermore, a single-center design increases the risk of selection bias. In particular, the concentration of more complex or advanced-stage patients in tertiary or referral centers may lead to a shift in the severity of the disease within the sample (spectrum bias). For these reasons, although single-center studies generally provide more controlled and homogeneous data, the generalizability of their findings is limited, and the results must be validated in different centers and among broader populations.

The relationship between the Tp-e interval and developing arrhythmia was not examined. It may not always be possible to reliably and consistently define and detect the development of arrhythmia. Particularly in situations where continuous monitoring is not available (e.g., patients admitted to general wards rather than intensive care units), there may be under-recording of arrhythmia episodes (misclassification bias). For this reason, we opted for mortality, which provides a more objective and comprehensive measure of outcome. Furthermore, the clinical significance of arrhythmia development alone may be heterogeneous. While some arrhythmias have a benign course, others may be fatal. This heterogeneity can make it methodologically challenging to treat arrhythmia as a single outcome and may complicate the interpretation of results. Furthermore, considering that arrhythmia may play a potential mediating role in the relationship between the Tp-e interval and mortality, the exclusion of this variable from the model limits the ability to distinguish whether the relationship is direct or indirect. The fact that arrhythmia development was not evaluated as an outcome both weakens the strength of causal inferences and limits the ability to interpret the study’s findings from a pathophysiological perspective.

There is a need for new studies on the relationship between Tp-e interval and intensive care triage, prediction of development of cardiac complications, risk stratification, and the sympathetic nervous system in both hemorrhagic and ischemic strokes.

## 5. Conclusions

The management of stroke patients is mostly done by focusing on neurological functions and etiology. Recent developments show that the brain–heart axis is deeper, more complex, and more mysterious than we thought. This syndrome, which can be named cardiocerebral syndrome, waits to be investigated with different subtypes. Therefore, stroke patients should be carefully examined and followed not only from a neurological perspective but also in terms of possible cardiovascular effects. New monitoring methods, risk stratification, as well as diagnostic and prognostic markers, should be developed for possible cardiovascular effects. The brain–heart axis and management of possible complications should be included in the curriculum of residency training. We suggest that the Tp-e interval can play an important role in the management of this syndrome.

## Figures and Tables

**Table 1:** Demographic characteristics of patients.

Age, years, median (IQR)	72.3 (10.5)
Sex, *n* (%)	
Male	49 (45.4)
GCS, median (IQR)	14 (6)
**Stroke type, *n* (%)**	
Infarct	89 (82.4)
Hemorrhage	19 (17.6)
CAD, *n* (%)	22 (20.4)
DM, *n* (%)	46 (42.6)
Hypertension, *n* (%)	79 (73.1)
CRF, *n* (%)	7 (6.5)
**Lesion location, *n* (%)**	
Right hemisphere, *n* (%)	60 (55.6)
Insular cortex involvement, *n* (%)	50 (46.3)
Thalamus involvement, *n* (%)	19 (17.6)
Supratentorial location, *n* (%)	81 (75)
Volume, cm^3^, median (IQR)	12.4 (46.4)
Systolic blood pressure, mmHg, median (IQR)	155.7 (32.1)
Diastolic blood pressure, mmHg, median (IQR)	85.9 (15.3)
Pulse/min median (IQR)	81.9 (22)
Troponin, median (IQR)	0.04 (0.06)
Ejection fraction, median (IQR)	54.6 (11.05)
ST elevation, *n* (%)	39 (36.1)
ST depression, *n* (%)	20 (18.5)
T inversion, *n* (%)	34 (31.5)
QTV_2_, ms, median (IQR)	343 (41)
QTc V_2_, ms, median (IQR)	382 (44)
Tp-eV_2_, ms median (IQR)	92 (16)
QTV_5_, ms, median (IQR)	354 (45)
QTcV_5_, ms, median (IQR)	393 (43)
Tp-eV_5_, ms, median (IQR)	92 (17)
Pulse-24.h/min, median (IQR)	84 (21)
ST elevation-24 h, median (IQR)	18 (16.7)
ST depression-24 h, median (IQR)	10 (9.3)
T inversion-24 h, median (IQR)	20 (18.5)
QTV_2_-24 h, ms, median (IQR)	345 (44)
QTc V_2_-24 h, ms, median (IQR)	388 (37)
Tp-eV_2_-24 h, ms, median (IQR)	84 (16)
QTV_5_-24 h, ms, median (IQR)	349 (46)
QTcV_5_-24 h, ms, median (IQR)	390 (32)
Tp-eV_5_-24 h, ms, median (IQR)	81 (18)
In-hospital mortality, *n* (%)	37 (34.3)

GCS: Glasgow Coma Score; IQR: interquartile range; CAD: coronary artery disease; DM: diabetes mellitus; CRF: chronic renal failure.

**Table 2:** Unadjusted and adjusted OR for in-hospital mortality in stroke patients.

	Unadjusted OR (95% CI)	*p* Value	Adjusted OR (95% CI)	*p* Value
SBP	0.99 (0.98–1.000)	0.34		
DBP	0.96 (0.93–0.99)	0.02	0.96 (0.93–1.000)	0.05
Pulse/min	1.00 (0.98–1.02)	0.47		
CAD	1.3 (0.51–3.5)	0.53		
Troponin	0.73 (0.46–1.14)	0.17		
Ejection fraction	1.03 (0.98–1.38)	0.23		
ST elevation	0.68 (0.29–1.61)	0.38		
ST depression	1.4 (0.51–3.9)	0.49		
T inversion	1.07 (0.45–2.5)	0.87		
QTV_2_	0.99 (0.97–1.002)	0.09		
QTcV_2_	0.99 (0.97–1.004)	0.24		
Tp-eV_2_	0.96 (0.93–0.99)	0.008	0.96 (0.93–0.99)	0.008
QTV_5_	0.99 (0.98–1.004)	0.25		
QTcV	0.99 (0.97–1.005)	0.41		
Tp-eV_5_	0.98 (0.96–1.005)	0.12		

CAD: coronary artery disease; SBP: systolic blood pressure; DBP: diastolic blood pressure.

## Data Availability

The data supporting this study are available from the corresponding author upon reasonable request. The data are not publicly accessible due to privacy and ethical considerations.
